# Diversity and Plasticity of Virulent Characteristics of *Entamoeba histolytica*

**DOI:** 10.3390/tropicalmed8050255

**Published:** 2023-04-29

**Authors:** Yasuaki Yanagawa, Upinder Singh

**Affiliations:** 1 Department of Microbiology and Immunology, Stanford University School of Medicine, Stanford, CA 94305, USA; yasu0730@stanford.edu; 2 Division of Infectious Diseases, Department of Internal Medicine, Stanford University School of Medicine, Stanford, CA 94305, USA

**Keywords:** intestinal parasite infection, *Entamoeba histolytica*, pathogenesis, virulence factor, diversity, animal model, asymptomatic infection

## Abstract

The complexity of clinical syndromes of amebiasis, caused by the parasite *Entamoeba histolytica*, stems from the intricate interplay between the host immune system, the virulence of the invading parasite, and the surrounding environment. Although there is still a relative paucity of information about the precise relationship between virulence factors and the pathogenesis of *Entamoeba histolytica*, by accumulating data from clinical and basic research, researchers have identified essential pathogenic factors that play a critical role in the pathogenesis of amebiasis, providing important insights into disease development through animal models. Moreover, the parasite’s genetic variability has been associated with differences in virulence and disease outcomes, making it important to fully understand the epidemiology and pathogenesis of amebiasis. Deciphering the true mechanism of disease progression in humans caused by this parasite is made more difficult through its ability to demonstrate both genomic and pathological plasticity. The objective of this article is to underscore the heterogeneous nature of disease states and the malleable virulence characteristics in experimental models, while also identifying persistent scientific issues that need to be addressed.

## 1. Introduction

*Entamoeba histolytica* (*E. histolytica*), which is a protozoan parasite, is responsible for causing amebiasis, which is a disease that can affect anyone [1] but is more prevalent in individuals residing in tropical regions with subpar sanitation facilities [2,3]. The infection can occur through ingestion of contaminated food or water, or through poor hygiene practices. This parasite has also been widely transmitted via sexual contact in developing areas [4,5]. Symptoms range from mild diarrhea to severe abdominal pain and bloody diarrhea [6]. In some cases, the infection can spread to other organs, leading to a life-threatening condition called invasive amebiasis [7]. *E. histolytica* infection is a public health issue not only in developing countries with poor sanitation, but also in developed countries, where it is a leading cause of morbidity and mortality [8,9,10]. Although most individuals infected with *E. histolytica* may not exhibit any symptoms, they can still transmit the infection, making it important to understand the pathogenesis of the parasite, which is currently not well-known due to limited data. Establishing laboratory data from a range of *E. histolytica* strains is essential to comprehending the breadth of the disease; however, there have been longstanding technical barriers to achieving this goal [11]. *E. histolytica* exhibits a high degree of genetic diversity [12]. *E. histolytica* may display a modest degree of nucleotide diversity; however, the genetic variation in gene family content and copy numbers can vary significantly between sequenced genomes, highlighting the parasite’s potential to evolve and adapt to different environments [13]. This diversity is mainly attributed to the presence of various strains of the parasite, each with distinct genetic characteristics [14]. Ongoing research is crucial to comprehending the pathogenesis of amebiasis, since the diversity of *E. histolytica* emphasizes the need to study genetic and biological factors that contribute to the parasite’s variation. In addition to the genetic diversity, the plasticity of *E. histolytica* is essential for its survival and pathogenesis [15]. This plasticity allows the parasite to evade the host immune system and maintain infection, making it challenging to develop effective treatments [16].

## 2. Variety of *E. histolytica* Infectious Disease Status

### 2.1. Symptomatic Infection

**Figure 1:**
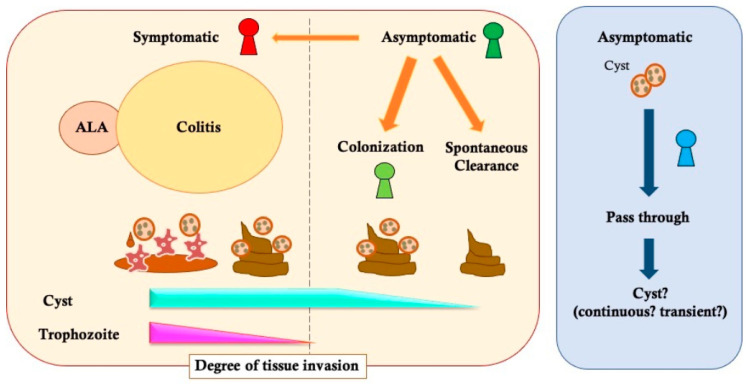
An Overview of *E. histolytica* infection potential in humans. Disease caused by *Entamoeba histolytica* infection can be classified based on degree of tissue invasion. Examination of tissues under a microscope can reveal characteristic damage caused by infection regardless of presence or absence of symptoms. Symptomatic patients often excrete watery or loose diarrhea with trophozoites and/or cysts of parasite. Sometimes, there are cysts detected in asymptomatic individuals’ stool. It is not yet clear if a transient infection could result in histological damage on mucosa and act as a potential source of secondary infection.

*E. histolytica* infection occurs when a person swallows the cyst forms from contaminated water or food. After ingestion of cysts, they pass through the gastrointestinal route without any symptoms until reaching the cecum site in colon. The excystation from non-invasive cyst forms to invasive forms of trophozoites is induced through a binding process in the colon epithelium. Symptomatic disease occurs when trophozoites invade the colon mucosa, destroy the structure of intestinal surface and lumen, and cause intestinal inflammation and ulceration [17,18]. Although a large number of infections remain asymptomatic, there are various types of symptomatic presentations (Figure 1). It is traditionally said that 10% of infected cases show symptoms [6]; however, the real percentages of symptomatic patients in the diagnosed-*E. histolytica* infection are varied, ranging from <1% to 92% in the targeted populations, and depend on the diagnostic tools [19,20,21,22]. The previous report showed that the parasite can be spontaneously eradicated within a mean period of 8.6 months without any treatment [21]; therefore, the symptomatic percentage in *E. histolytica*-infected patients may be influenced by the timing of specific diagnostic procedures and the clinical severity of amebiasis.

The majority of symptomatic infections in patients are caused by amebic colitis, while the minority of manifestation/complications are caused via the progression of extraintestinal diseases, including liver abscess. In symptomatic individuals, the presence of blood and/or mucus in stool, including hematochezia, abdominal pains including cramps, and diarrhea, are major symptoms, although it is said that dysentery and tenesmus are the specific symptoms for amebic colitis [23,24,25]. Even though *E. histolytica* can cause infectious colitis in humans, there are a variety of severities for each person, ranging from an intermittent mild course to a life-threatening fulminant disease. In particular, in groups containing individuals who present with subacute/chronic gastrointestinal symptoms, some individuals are initially misdiagnosed with Inflammatory bowel disease (IBD) [25]. The main reasons for the wrong diagnosis are as follows; (1) presentation (abdominal symptoms, intermittent episodes, and chronic time course) of amebic colitis is often similar to symptoms of IBD; (2) non-specific endoscopic findings for physicians who did not recognize the parasite infection as a differential diagnosis; and (3) non-specific pathological findings based on the hematoxylin and eosin stain, which sometimes make it technically hard for all physicians bar well-experienced laboratory staff to distinguish these cells from inflammatory cells [18,26,27,28]. This misdiagnosis could lead to the inappropriate use of corticosteroid therapy and induce the exacerbation of amebic colitis to severe status. Shirley reported, in a systematic review of fulminant amebic colitis, that 58% (14 of 24 trial patients) were treated with corticosteroids due to initial misdiagnosis with inflammatory colitis; half underwent inappropriate surgical intervention [29]. Therefore, physicians and laboratory staff should consider parasite infection when treating patients showing subacute/chronic clinical gastrointestinal symptoms and non-specific laboratory findings in specimens because there is currently no complete single tool for diagnosis.

Amebic extraintestinal infection consists of mainly liver abscess; other presentations can include pleuropulmonary disease, brain abscess, peritonitis, pericarditis, genitourinary disease, and inguinal lymphadenitis [30,31,32,33,34]. Moreover, there were immune-related complications associated with *E. histolytica* infection, such as immune complex glomerulonephritis [35].

### 2.2. Asymptomatic Infection

4–10% of asymptomatic individuals infected with *E. histolytica* develop disease over a year [36]. The pathogenesis of the asymptomatic infection still unclear; however, there are several host factors reported to affect the disease: HLA subtype [5,37,38,39,40], intestinal IgA level [36,41], and gut microbiota [42]. The majority of asymptomatic individuals are diagnosed in clinical studies for epidemiology or diagnosis for *E. histolytica*, or accidental medical diagnosis during routine medical check-ups or other procedures, including colorectal cancer screening (Figure 1) [4,5,43]. Although some past cases were accidentally diagnosed via the stool testing for cyst passers, the common stool examinations generally lack sufficient sensitivity, except for the PCR method [17,44]. Even the antigen detection method, including ELISA, which is globally used in many settings, has a risk of not detecting the parasite, especially for cysts containing specimens [4]. This method can be affected by proteomic analysis, given that the cyst stage has a specific proteome that is never detected in the trophozoite stage [45]. Therefore, the endoscopy examination is the most reliable tool for early detection of asymptomatic infection [26]. Interestingly, the distribution of mucosal abnormality via *E. histolytica* in colon is a more common characteristic in asymptomatic individuals compared with symptomatic patients. In asymptomatic individuals, the mucosal abnormality is mainly limited in cecum and ascending colon; however, in symptomatic patients, there are multiple lesions found in the whole colon [17,43]. Sigmoidoscopy could fail to detect the mucosal lesions and lead to incorrect diagnosis for *E. histolytica* infection [21]. This problem may be caused by the limitation of observational parts in the colon when it is performed on via sigmoidoscopy. Certainly, in some cases, the parasite does not suffer from any parasite-related bowel complaints and can be eradicated spontaneously [20]. However, the appropriate treatment is currently warranted in all cases of infection with *E. histolytica*, even in asymptomatic individuals, because there a lot of personal factors influencing the transformation of the silent disease into more severe disease, such as age, pregnancy, corticosteroid, malignancy, malnutrition, and alcoholism [1,27,29].

### 2.3. The Diagnostic Gap about “Cyst Carrier”

Currently, there are two different axes to diagnose *E. histolytica* infection: one axis is based on ‘symptomatic’ vs. ‘asymptomatic or colonization’ infection in the individual level, while the other axis is based on ‘invasive’ vs. ‘non-invasive’ infection associated with inflammation in the tissue level. The word ‘invasive’ infection is used in many studies as having the same definition as ‘symptomatic’ infection because those symptoms are directly and indirectly caused by invasion of parasite and tissue damage by *E. histolytica* infection (Figure 1) [1,6,46,47,48]. In the stage of *E. histolytica*, the trophozoites have the potential to survive and damage human tissues, and the cyst has been recognized as a non-pathogenic infectious form that spreads to other hosts [6]. In clinical settings, the cyst form has often been detected in stools of asymptomatic or colonization individuals, and can be a diagnostic trigger for these individuals [4,49,50]. Thus, there are some reports that mostly recognized those cyst carriers as identical to ‘asymptomatic or colonization’ infection by *E. histolytica* [21,37,51]. These researchers believed that there were no clear tissue damages leading to invasive infection happened in asymptomatic individuals. However, as reports about asymptomatic cases diagnosed via endoscopy are compiled, new facts are coming to light about the pathogenesis of asymptomatic infection [5,17,23]. There are macroscopically visible lesions on the human colon detected via endoscopy, regardless of any cysts detected in stools [17]. Pathological approaches found host cell death, destruction of mucus layer, and infiltration of inflammatory cells in the lesion sites. In fact, we reported the exacerbation by cases of asymptomatic chronic infection to acute colitis [42]. These facts indicate that *E. histolytica* can even cause tissue damage at the mucus level in asymptomatic individuals. In other words, the histological ‘invasive’ infection happens both in symptomatic and asymptomatic infection. Therefore, when the patients infected with *E. histolytica* are classified, it would be more appropriate to emphasize symptoms rather than invasive appearance.

At the clinical scene of treatment, there are issues which concern physicians’ selection of the appropriate drugs for *E. histolytica*. It is currently recommended that standard treatments are metronidazole plus a luminal agent for invasive amebiasis, and only a luminal agent for non-invasive colonization [6,52]. As mentioned above, it has been clear that there are histological ‘invasive’ phenomena, including the multiple mucus damage and pathological inflammatory reactions already induced in the human colon, such as progressive-to-severe disease in even asymptomatic individuals. Furthermore, paromomycin, which is one of the luminal agents, is not systemically absorbed, being 100% excreted in an unchanged form from stool [53,54]. Taken together, these results suggest that the best therapy for asymptomatic individuals who present the mucus lesions detected via endoscopy remains undetermined. Paromomycin monotherapy may be a good selection [49], though it is unclear that the drug will adequate for all asymptomatic individuals. Further investigations are required to determine appropriate therapy for asymptomatic *E. histolytica* infection.

## 3. Plasticity of *E. histolytica* Virulence

### 3.1. Attenuation and Reactivation of the Parasite Virulence through Animal Models

The degree of virulence of *E. histolytica* can be assessed experimentally on the basis of three criteria: the ability to induce liver abscesses in hamsters, cytopathic and cytotoxic effects in cultured mammalian cells, and erythrophagocytosis [55,56,57,58]. To investigate the virulence of *E. histolytica* in animal models, the liver abscess model in golden hamsters is used [59,60,61]. The experimental inoculation and induced liver abscess were analyzed for the various virulent factors of vaccine development [62,63,64,65]. However, after isolating the clinical *E. histolytica* strains from the patients and maintaining subcultures, most *E. histolytica* strains can lose their potential to induce liver abscess in hamsters at a relatively early stage. Bos and Hage reported that some strains derived from symptomatic and asymptomatic decreased the virulence within two to fifteen weeks [66]. To maintain its virulence to induce liver abscess in hamsters, it is necessary to regularly repeat the liver passages through animal models [67]. Moreover, even in the same *E. histolytica* cell lines, it is known that there are phenotypic variations of the virulence in gerbils and mice. Meyer et al. reported that through the animal models using HM-1 laboratory strain as a virulent strain, which was isolated from amebic colitis with dysentery in 1967, the different *E. histolytica* cell lines revealed significantly differences in their pathogenicity and were divergent from clones derived from one cell lines [68]. In the process of isolation and in vitro culture, the *E. histolytica* clinical strains must pass through the several conditions: xenic culture with bacteria, monoxenic culture with *Crithidia fasciculata*, and axenic culture [69]. The laboratory strain NIH-200 showed the different virulence in rat models between axenic and xenic conditions [70]. It is well known that even the virulent *E. histolytica* HM-1 stain can lose virulence against animal model under long axenic culture conditions [71,72]. On the other hand, the virulent potential is reversible because it can be restored in axenic condition and increased after association with bacteria. Regardless of asymptomatic infection isolated from the original cases, some *E. histolytica* laboratory strains were reported to modify their virulence after association with E. coli serotype 055 or serotype 0115 strains [55,73]. In addition to the liver abscess models for hamsters, Fernandez-Lopez and et al. reported that the interaction with enteropathogenic E. coli could increase intense inflammation and pro-inflammatory cytokine genes in closed colonic loop models of mice [74]. These results demonstrate that the gut interaction may have a major role in modulating the severity and progression of *E. histolytica* infection in human.

### 3.2. Impacts of the Parasite’s Factors on the Diversity of Amoebic Infection

**Figure 2:**
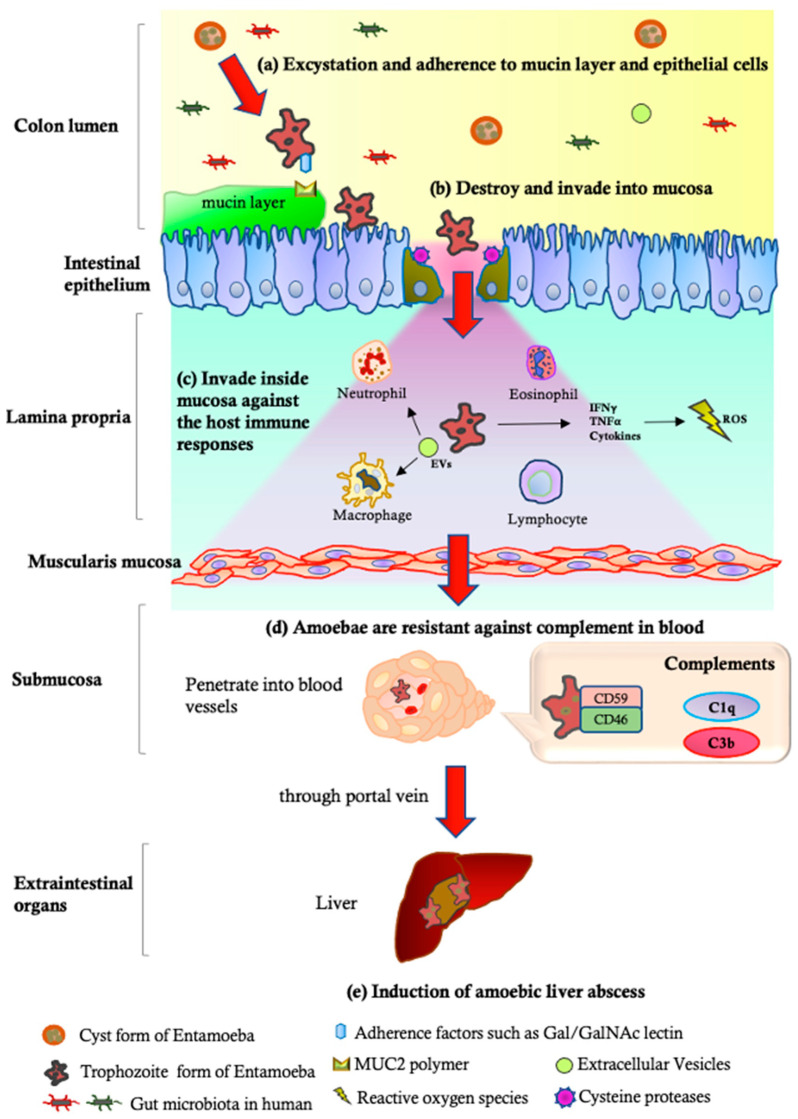
Steps of pathogenesis of *Entamoeba histolytica* invasive disease. (**a**) Cyst form of parasite is ingested by host and travels to terminal ileum region where it undergoes excystation, releasing trophozoites. Trophozoites then adhere to intestinal mucosa using surface proteins and glycoproteins. (**b**) Trophozoites use enzymes to destroy protective mucus layer of intestinal wall and penetrate mucosa. This act causes tissue damage and inflammation, leading to symptoms such as abdominal pain, diarrhea, and bleeding. (**c**) Parasite evades host immune system by changing surface antigens frequently, which prevents host antibodies from recognizing and attacking it. Additionally, it can suppress the host immune response and even kill immune cells. (**d**) Parasite is resistant to the complement system in blood, which is part of innate immune system that can destroy invading pathogens. *E. histolytica* can produce a glycoprotein that inhibits complement system, allowing it to evade immune attack. (**e**) In some cases, parasite can invade bloodstream and travel to other organs, such as liver, where it can cause abscesses. This can occur when trophozoites penetrate intestinal wall and enter portal circulation.

*E. histolytica* is generally recognized as a pathological intestinal parasite; on the other hand, *E. dispar* is well-known as a non-pathological burden. The morphological findings of *E. dispar* via microscope are identical to *E. histolytica*; however, there are genetic distinctions between the two organisms. For DNA analyses, the degree of identity within the orthologous sequence is about 95% for the coding regions and about 80% for the intergenic regions [75]. From the basic experiments and the diverse clinical findings of *E. histolytica*, it is believed that several steps are required to establish the parasite infection in the human body after ingestion of the cyst form (Figure 2, Table 1). Firstly, reaching the cecum lesion through gastrointestinal tracts, *E. histolytica* attaches the surface of mucin layer on colon mucosa. The process of excystation occurs in the end part of the small intestine known as the terminal ileum, which produces mobile and possibly invasive trophozoites from the cyst [76]. The parasite’s Gal/GalNAc lectin, which specifically attaches to carbohydrates galactose (Gal) and/or N-acetyl-D-galactosamine (GalNAc) on the host cell membrane, is mainly responsible for the adherence of trophozoites to intestinal epithelial cells [77]. The development of the trophozoite within the colon epithelium involves its attachment to mammalian cells using other various mechanisms, such as LPPG, or direct contact between amoeba and cells via specific proteins, such as KERP, STIRP, ADH112, Jacob lectin, and SREHP [77,78,79,80,81,82,83]. A striking characteristic of *E. histolytica* during the process of tissue invasion is its capacity to cause lysis of human cells and degrade the extracellular matrix (ECM). The extracellular matrix (ECM) is a complicated network of proteins that offers structural support to tissues. Due to its physical barrier function, the breakdown of ECM is often necessary to enable cell migration through tissues. Matrix metalloproteinases (MMPs) are the primary enzymes responsible for ECM degradation. In the colon explant model, it has been observed that the enzymatic actions of human matrix metalloproteinases (MMP-1 and -3) are responsible for modifying the collagen fibrillar structures during intestinal invasion by *E. histolytica* [84]. The ability of cysteine proteases (CP) to degrade ECM proteins makes them directly implicated in tissue invasion [85]. CP-A5 is the primary CP of *E. histolytica* involved in the pathogenic process, localizing on the surface of the amoebae, and has been found to be responsible for amebiasis, including human colon invasion and ALA formation [64,86]. CP-A5 activity is known to convert pro-MMP-3 into its active form, subsequently activating pro-MMP-1, leading to the degradation of collagen [87]. In addition to CPs, the amoebapores (AP), which are a family of three pore-forming peptides, have the capability to insert themselves into the membranes of both bacteria and eukaryotic cells, thereby creating pores that lead to the lysis of the targeted cells [88,89]. The introduction of pure APs to eukaryotic cells is known to cause necrosis and potentially apoptosis; however, even in the absence of AP-A, *E. histolytica* trophozoites can still induce inflammation and inflict tissue damage in colonic xenografts of infected humans [90]. Taken together, it is becoming clear that the major factors that induce amoebic colitis or liver abscess are distinct and represent separate pathological steps. Although host immune responses, including both innate and adaptive defenses, are strong against invasive *E. histolytica*, this parasite is still able to persist by developing evasion strategies against the immune system. Neutrophils and macrophages react to pathogens by generating reactive oxygen species (ROS) via the NADPH oxidase system, which act as a potent antimicrobial defense mechanism by damaging the pathogen’s cell membrane and DNA [91]. *E. histolytica* possesses two enzymes—iron-containing superoxide dismutase and NADPH:flavin oxidoreductase—which enable it to detoxify ROS by generating H_2_O_2_ [92]. Trophozoites of Eh use a surface protein called peroxiredoxin, which has potent antioxidant properties, to shield themselves from the reactive oxygen species of neutrophils [93]. Recent studies found that the extracellular vesicles (EVs), which are membranous bodies and produced by practically all living organisms from bacteria to humans, have a critical role in modulating the host immune reaction [94]. The absorption of amoebic EVs by neutrophils was proven through fluorescence, which yielded a significant decrease in the oxidative burst and neutrophil extracellular trap (NET) secretion [95]. After penetrating the blood flow, the complement system plays a critical role in protecting against parasites in the blood, helping to identify and eliminate them through a series of immune responses [96]. Activation of the host complement system, which results in the formation of the membrane attack complex (MAC), has the potential to lyse the *E. histolytica* trophozoites and, thus, prevent their dissemination into the extraintestinal space. *E. histolytica* has an ability to resist complement activation involving GalNAc, which has antigenic cross-reactivity with CD59 and can inhibit MAC-mediated lysis [97]. CPs are also capable of cleaving complement components [98], though the extracellular cysteine proteases can effectively degrade pro-inflammatory complement components C3a and C5a [99]. Moreover, *E. histolytica* can degrade secretory IgA and serum IgG in vitro, despite the fact that these immunoglobulins mediate adaptive immunity against the parasite [100,101]. Calreticulin (CRT), which was identified as the primary calcium-binding protein of the endoplasmic reticulum, has recently been reported as an inhibitor to activate the classical complement pathway [102]. As it passes through the innate and adaptive immune system in the blood, the resistant *E. histolytica* can reach extraintestinal organs, mainly the liver, via the portal vein. As amoebae first establish themselves in the liver parenchyma and tissue necrosis spreads, parasites interact with liver parenchymal cells, leading to the destruction of hepatocytes and activation of host immune cells [103]. The experimental animal models revealed the responsible roles of CPs and Gal/GalNAc lectin in ALA formation [104,105]. Recent research indicates that peroxynitrite (ONOO−) plays a substantial role in the formation of abscesses, reducing the importance of other mechanisms. Additionally, it appears that amoebas have a superior defense mechanism against ONOO− than mammals, namely the amebic thioredoxin system, including peroxiredoxin [106].

Compared with the virulent *E. histolytica* HM1:IMSS strain, the *E. histolytica* Rahman strain and *E. dispar* are recognized as avirulent strains because they are isolated from asymptomatic carriers and show low levels of cytotoxicity in vitro [79,107,108]. Additionally, it has been reported that the signals relating to many virulent factors against host immune response in *E. dispar* are generally absent or lower than *E. histolytica* HM1:IMSS strain. Earlier in vitro and in vivo investigations provide compelling evidence indicating that CPs play a crucial role in facilitating amoeba pathogenicity; however, the majority of CPs’ gene expressions are missing in *E. dispar* [109]. Although these two strains are utilized in many past studies focusing on the parasite virulent as a non-virulent control, it is necessary to reconsider the paradigm that exclusively identifies *E. histolytica* as the pathogen that can lead to serious damage in the large intestine and extraintestinal organs. Newly accumulated suggsets that some *E. dispar* strains have the potential to induce liver damage in experimental models [110,111,112,113,114]. These discrepancies in virulent characteristics suggests that, for the diverse amoebiasis, each pathological step requires a different mechanism, rather than a simple cascade. In fact, the severe amebiasis cases caused by *E. dispar*, such as amoebic liver abscess, have never been reported, even though some *E. dispar* strains could produce them in artificial animal models. This fact implies that the unique virulent characters of *E. dispar* meet the necessary condition to induce liver damage once the parasite reaches the liver, and are missing required elements to progress deeply inside the mucosa and pass through the intestinal tract [115,116]. Future research is needed to elucidate the role of each virulence factor for specifically establishing amebiasis.

**Table 1:** Major parasite factors relating to each pathogenic step in *E. histolytica*; a comparison of pathological and non-pathological strains (HM-1:IMSS and Rahman) and non-virulent species *E. dispar*.

Pathogenetic Steps	Parasite			Reference
	*E. histolytica*		*E. dispar*	
	Pathological Strain, HM1:IMSS	Non-Pathological Strain, Rahman		
(a) Excystation and adherence to mucin layer	Gal/GalNAc-specific lectins [77], LPPG [77], KERP1 and KERP2 [78], STIRP [79], ADH112 [80], Jacob lectin [82], SREHP [83]	Deficiency in the expression of the 35 kDa subunits of the Gal-lectin complex [117]	Decreased surface lectin; deficiencies in immunodominant 1G7 epitopes [118]	[77,78,79,80,82,83,117,118]
(b) Destruction and invasion into mucosa	CPs [64,85,109], AP [88], CPADH [119], Phospholipase [120], and ROMs [121]	Decreased ESP (eg. Carbohydrate metabolizing enzymes) [122]; decreased CP activity in response to degradation [87]	Rarely expression levels of CP-1 and CP-5 [109]; decreased CP activity in response to degradation [87]	[64,85,88,109,119,120,121]
(c) Invasion inside the mucosa and escape from the host immune responses	Gal/GalNAc lectin [123], ROM1 [124], CPs [125], LPG [126], Phosphatidylcholine [120], HSPs [127], Superoxide dismutase [128], NADPH:flavin oxidoreductase [129], and MIF [130]	Decreased mRNA levels of MUC2, poly-lg receptor, and SOCS1 [131]	No induction of NET formation and ROS production [132]	[120,123,124,125,126,127,128,129,130,131,132]
(d) Amoebic resistance to complements	CRT 1 [102], Gal/GalNAc lectins [97], LPG [133], and Secretory IgA proteases [134]	Sensitive to complement [135]	Decreased expression of CRT levels [102]; near-total absence of LPG-like glycoconjugates to protect against complements [116]; highly sensitive to C9 complements [115]	[97,102,115,116,133,134,135]
(e) Induction of amoebic liver abscess	AP [89,90], Gal/GalNAc lectins [117,136], CPs [64,134], Phospholipases [137,138], Peroxynitrite [106]	ND	Ability to induce liver abscess in inoculated animals [110,111,112,113,114]	[64,89,90,106,110,111,112,113,114,117,134,136,137,138]

Abbreviations: Gal/GalNAc, Galactose/N-acetylgalactosamine; KERP, Lysine(K)- and glutamic acid(E)-rich protein; ADH, protein with an adherence domain; CPADH, complex of CP112 and ADH112; STIRP, serine-, threonine-, and isoleucine-rich proteins; ROM, rhomboid proteases; ESP, excretory-secretory products; CP, cysteine proteases; AP, amoebapores; LPG, lipophosphoglycan; HSP, heat shock proteins; EV, extracellular vesicle; MUC, mucin; SOCS, suppressive of cytokine signaling; NET, neutrophil extracellular trap; ROS, reactive oxygen species; CRT, calreticulin; MIF, macrophage migration inhibitory factor; ND, no data.

## 4. Variation in *E. histolytica* Strains

### 4.1. The List of Major E. histolytica Laboratory Strains Studied until Now

Laboratory strains of *E. histolytica* are typically derived from clinical isolates through the axenization process, and maintained via culturing the parasite in axenic conditions [69]. These strains have been extensively studied and characterized, and are used to investigate the biology, pathogenesis, and drug sensitivity of *E. histolytica* [14,139,140]. They have been shown to exhibit a range of phenotypic variations, including differences in virulence, motility, phagocytosis, and antigenicity [57,81,141,142]. Some commonly used laboratory strains of *E. histolytica* include HM-1:IMSS, Rahman, and HK-9, which have been widely used in research studies over the years. In particular, the HM-1:IMSS, when isolated from a rectal ulcer of a patient with amoebic dysentery in 1967, is used in research as a pathological strain. Compared with the Rahman strain, as a non-pathological strain, the major virulence factors of amoebiasis were identified based on in vitro, in vivo, and ex vivo models [81,143]. Interestingly, the pathological HM1:IMSS was not always able to produce the liver abscess in the animal model, unless a genetic mutation to suppress the host immune system was present [71,73,144]. In the colitis model, only 67% of the inoculated rats could be induced to generate the inflammatory lesion [71]. Even though the pathophysiology of amoebiasis is a complex interplay between several factors, such as host immune responses and the cultural condition of *E. histolytica*, certain virulent strains exhibit similar pathogenic characteristics in both humans and animal models (Table 2). Certainly, some virulent strains, such as HM-1:IMSS, NIH-200, DC, SF, and EGG, have demonstrated the capability to induce liver abscess in hamsters. On the other hand, paradoxical strains are reported. Despite being isolated from asymptomatic carriers, the strains SAW:1734, CDC:0784:4, and BF-841 have been found to possess the capability to induce liver abscess in hamsters [71,145]. Since host immune response plays a significant role, it is more challenging to maintain a dysentery model using amoeba than a liver abscess model [146]. As evident from the Table 2 of axenically cultured strains, since amoeba isolates exhibit diverse pathogenicity in both humans and animal models, careful interpretation is necessary while investigating amoebic pathogenic factors.

### 4.2. Genetic Variation among the Main Laboratory Strains

The draft genome sequence of *E. histolytica* (strain HM-1:IMSS) was first published and analyzed in 2005, with subsequent reassembly and reannotation in later studies [161,162,163]. The genome is composed of 35 M base pairs of DNA, which have a high AT content of approximately 75% and are gene-rich, with around half of the assembled sequence predicted to be coding, containing 8734 protein-coding genes. *E. dispar* has a genome assembly of similar size, with 22.9 M base pairs of DNA in 3312 scaffolds [164]. Its AT content is also similar to *E. histolytica*, being approximately 76.5%, and it has a similar proportion of predicted coding sequence, with 8749 annotated genes. The genomic structure and arrangement of *Entamoeba* are not well understood. It is currently unclear whether there is a naturally occurring number of chromosomes in this organism and whether it is haploid or polyploid, despite estimates for both options [163,165].

A DNA microarray, constructed using a clone library that included 2110 unique genes, was utilized to compare the variation in genomic DNA among four *E. histolytica* laboratory strains (HM-1:IMSS, Rahman, HK9, and 200:NIH) and two *E. dispar* strains (SAW760 and SAW1734) [166]. All strains were found to have unique genetic fingerprints due to the identification of divergent genetic loci. By comparing divergent genetic regions, it was able to distinguish between *E. histolytica* and *E. dispar*, identify novel genetic regions that can be used for strain and species typing, and discover several genes that are limited to virulent strains. Among the four *E. histolytica* strains, the strain with attenuated virulence was the most divergent and phylogenetically distinct, indicating that genetic subtypes of *E. histolytica* may play a role in the variability in clinical outcomes observed. Based on the transcriptional profiling using DNA microarray, 29 genes had lower expression levels in both *E. histolytica* Rahman and *E. dispar* SAW760 compared to expression in the virulent *E. histolytica* HM-1:IMSS [167]. Those decreased genes had roles in pathogenesis or stress response.

### 4.3. Genetic Differences and Variable Patterns of Gene Expression between Laboratory and Clinical Strains

Researchers undertook several transcriptome-level studies to gain insights into the pathology and biology of *E. histolytica* [13,15,168,169,170]. These investigations have encompassed various aspects, such as analyzing gene expression patterns in virulent and avirulent strains, under different stress conditions, and after anti-amoebic drug treatments. Additionally, gene expression changes during the process of encystation have also been studied [171]. Through gene expression analysis, significant differences were observed in the molecular factors associated with virulence between the HM-1:IMSS and Rahman strains [93,143,167]. By comparing the transcriptome of the two strains using a custom 70 mer oligonucleotide-based microarray that covered the majority of the *E. histolytica* HM-1:IMSS genome, significant disparities between the two strains were observed. In particular, distinct gene expression patterns were identified for cysteine proteinases, AIG family members, and lectin light chains [93]. The virulent strain exhibited a significant upregulation of genes associated with carbohydrate metabolism, and, when the glycoside hydrolase (ß-amylase) was downregulated, the virulence of HM-1:IMSS was abolished, as confirmed through the absence of mucus depletion and tissue invasion [143]. This finding implies that in low-glucose conditions in the colon, virulent *E. histolytica* may efficiently utilize host mucus glycans as a carbon source, which triggers intestinal amoebiasis. However, it remains unclear whether *E. histolytica*’s ability to use host mucus glycans as a carbon source determines its invasive potential. Moreover, the cell line of HM-1:IMSS can lose or maintain the virulence potential, as determined through abscess formation in animal model, after subculturing for long time. A comparison of the transcriptomes of two *E. histolytica* cell lines—HM-1:IMSS-A and HM-1:IMSS-B—with different abilities to induce liver abscesses revealed that only 19 genes showed a differential expression of five-fold or greater [172]. Among these genes, three rab7 GTPases were expressed more abundantly in the non-pathogenic cell line, while AIG1-like GTPases were more highly transcribed in the pathogenic cell line. Weber et al. investigated the transcriptomic changes of a single virulent amoebic strain in four different contexts: hamster liver abscess, human colon explants, long-term cultured virulence-attenuated cells, and short-term cultured trophozoites [15]. The study revealed significant transcriptome changes in virulent parasites upon contact with human colon explants, suggesting that the activity of glycosylase, cytoskeleton, and DNA repair mechanisms were crucial in amoebic intestinal invasion. The long-term cultured parasites showed increased proteasome activity and downregulation of translational machinery (tRNA synthetases), which likely altered the gene expression program. In a study comparing pathological strains (KU50 isolated from amoebic colitis and KU27 isolated from asymptomatic carrier), it was discovered that AIG1 family protein is a key factor in the adherence of the pathogen to host cells [173]. The lack of a particular AIG1 family gene (EHI_176590) in the genome of the KU27 strain was found to be linked to the formation of liver abscess in animal models, as its expression was associated with this pathology. Comparative studies between HM1:IMSS and other clinical strains of *E. histolytica* revealed the presence of several virulent factors associated with liver abscess formation in hamsters, including molecules involved in oxidative stress response (peroxiredoxin and thioredoxin), nitrogen compound biosynthesis, Gal/GalNAc lectin subunit Igl1, serine-rich *E. histolytica* protein (SREHP), and the pore-forming peptide amoebapore A precursor. [174]. Furthermore, the study revealed two significant observations regarding the pathogenicity of *E. histolytica*: (1) the continuous exposure to environmental stress elevates its virulence through causing alterations in gene expression; and (2) the genetic changes responsible for liver abscess formation may vary among different strains and are not always consistent.

## 5. Conclusions and Future Directions

The mechanisms by which *E. histolytica* causes disease in the host are regulated using multiple host and parasite variables, including the genetic makeup of the parasite, the host immune response, and environmental factors. Genetic diversity in parasite strains plays a significant role in determining the pathogenic potential of the parasite. *E. histolytica* exhibits a high degree of genetic diversity, with multiple strains and subtypes identified and associated with variable clinical outcomes. From the previous studies, it is clear that the major virulence factors, such as amoebapore, Gal/GalNAc lectin, and cysteine proteases, have critical impacts on the pathogenesis of *E. histolytica*. However, *E. histolytica* displays versatility in its ability to adapt to various environmental conditions and exhibit differential impacts on the human host. The pathogenicity of *E. histolytica* is a complex phenomenon that cannot be explained using a single pathway. Given the variability in virulence potential, it is crucial to focus greater research on clinical strains recently isolated from human infections to identify common critical processes relevant to adaptation of the parasite to the human host, as well as the variable clinical outcomes that can occur.

## Figures and Tables

**Table 2:** Virulence characterization of 18 *E. histolytica* laboratory strains cultured in axenic conditions.

Strain	Clinical Status in Human	Virulence				Reference
		Liver Abscess Model	Animal Model	Colitis Model	Animal Model	
HM-1:IMSS	Amebic colitis with dysentery [147]	Attenuated virulence	0~100% in wild type of hamsters [71,73]; 100% in SCID mice [144]	Attenuated virulence	67% in wild type of rats [71]	[71,73,144,147]
Rahman	Asymptomatic [71,108]	Avirulent	0% in wild type of hamsters [71]	Avirulent	0% in wild type of mouse [93,143]	[71,93,143]
HK9	Amebic colitis with dysentery [148]	Avirulent	0% in wild type of hamsters [149]	Avirulent	0% in wild type of guinea pigs and germfree rats [149,150]	[148,149,150]
NIH-200	Amebic colitis with dysentery [151]	Attenuated virulence	0~100% in wild type of hamsters [71]	Attenuated virulence	0% in wild type of hamsters, guinea pigs, and rats [70,150,152]	[70,71,150,151,152]
ABRM	Amebic colitis with rectal abscess [150]	N.D	N.D	Avirulent	0% in wild type of guinea pigs [150]	[150]
ICB-CSP	Amebic colitis with dysentery [73]	Avirulent	0% in wild type of hamsters [58,73]	ND	ND	[58,73]
ICB-462	Asymptomatic [73]	Attenuated virulence	25% in wild type of hamsters [58,73]	ND	ND	[58,73]
ICB-32	Asymptomatic [73]	Avirulent	0% in wild type of hamsters [58,73]	ND	ND	[58,73]
ICB-RPS	Asymptomatic [73]	Avirulent	0% in wild type of hamsters [58,73]	ND	ND	[58,73]
DC	Amebic colitis with dysentery [153]	Virulence	87% in wild type of hamsters; 0% in wild type of guinea pigs [153]	Attenuated virulence *	29% in wild type of hamsters [154]	[153,154]
SF	Amebic colitis with dysentery [153]	Virulence	100% in wild type of hamsters; 0% in wild type of guinea pigs [153]	Attenuated virulence *	58% in wild type of rats; 100% in wild type of guinea-pigs [155]	[153,155]
SAW:1734	Asymptomatic [71]	Attenuated virulence	60% in wild type of hamsters [71]	Avirulent	0% in wild type of rats [71]	[71]
CDC:0784:4	Asymptomatic [71]	Attenuated virulence	50% in wild type of hamsters [71]	Avirulent	0% in wild type of rats [71]	[71]
SAW:408	Amoebiasis (details unavailable)	ND	ND	Virulent *	100% in athymic rats; 80% in Wister rats [156]	[156]
EGG	Amebic colitis with dysentery and amebic liver abscess (details unavailable)	Virulent	100% in wild type of hamster [110]	Virulent	100% in C57BL/6CD^-/-^ mouse [157]	[110,157]
KU27	Asymptomatic [158]	Avirulent	0% in wild type of hamster [159] (details unavailable)	ND	ND	[158,159]
BF-841	Asymptomatic [145]	Virulent	Ability in wild type of hamster [145] (details unavailable)	ND	ND	[145]
IP-106	Fulminating amebic colitis with dysentery [160]	Virulent	100% in wild type of hamster [160]	Attenuated Virulent	50% in wild type of hamster [160]	[160]

* Maintained with associated bacteria. Abbreviations: ND, no data; SCID, severe combined immune deficiency.

## Data Availability

Not applicable.
